# Whole-Neck Non-Contrast-Enhanced MR Angiography Using Velocity Selective Magnetization Preparation

**DOI:** 10.3390/tomography9010006

**Published:** 2022-12-29

**Authors:** Chan Joo Park, Seung Hong Choi, Jaeseok Park, Taehoon Shin

**Affiliations:** 1 Division of Mechanical and Biomedical Engineering, Ewha Womans University, Seoul 03760, Republic of Korea; 2 Graduate Program in Smart Factory, Ewha Womans University, Seoul 03760, Republic of Korea; 3 Department of Radiology, Seoul National University Hospital, Seoul National University College of Medicine, Seoul 03080, Republic of Korea; 4 Department of Biomedical Engineering, Sungkyunkwan University, Suwon 16419, Republic of Korea; 5 Department of Intelligent Precision Healthcare Convergence, Sungkyunkwan University, Suwon 16419, Republic of Korea

**Keywords:** non-contrast-enhanced MRA, neck angiography, velocity-selective preparation pulse sequence

## Abstract

This study aimed to optimize velocity-selective magnetic resonance angiography (VS-MRA) protocols for whole-neck angiography and demonstrate its feasibility in healthy subjects with comparisons to clinical 3D time-of-flight (TOF) angiography. To help optimize VS-MRA protocols, 2D phase-contrast (PC) flow imaging and 3D B_0_ and B_1_ field mappings were performed on five healthy volunteers. Based on these measurements, a slab-selective (SS) inversion preparation was applied prior to a VS saturation preparation to further suppress venous blood, while the VS preparation pulse was designed with compensation for field offsets. VS-MRA and 3D TOF were performed on six healthy subjects, and relative contrast ratios (CRs) between artery and muscle signals were calculated for twenty arterial regions for comparisons. The pre-compensated VS pulse improved the visualization of the subclavian arteries and suppression of background tissues, which involved large B_0_ and B_1_ field errors. The combination of SS and VS preparations effectively suppressed venous blood. While the relative CR values were 0.78 ± 0.08 and 0.72 ± 0.10 for VS-MRA and 3D TOF, respectively, over the twenty segments, VS-MRA outperformed 3D TOF in visualizing arterial segments of a small size or with a horizontal orientation, such as subclavian, facial, and occipital arteries. The proposed neck VS-MRA with the field-error-compensated VS preparation combined with the SS preparation is feasible and superior to 3D TOF in visualizing small and/or horizontally oriented arterial segments.

## 1. Introduction

Non-contrast-enhanced (NCE) magnetic resonance angiography (MRA) is an ideal approach for safe imaging of the arteries, as it eliminates invasive operations, ionizing radiation, and the side effects associated with contrast agents [1,2,3,4]. A large variety of NCE MRA methods have been developed, among which time-of-flight (TOF) imaging [5,6], slab-selective inversion recovery (SS-IR) imaging [7,8], and quiescent interval single-shot (QISS) imaging [9,10] are now available in clinical practice. In particular, for carotid and cerebral arteries, 2D or 3D TOF imaging is widely used as a primary imaging tool or a supplementary NCE option for patients with renal dysfunction. TOF offers a robust visualization of vessels of a large size and with a superior-to-inferior (S-I) orientation, but often shows suboptimal performance for vessel segments of a small size or with an in-plane orientation due to flow saturation effects [2].

Velocity-selective (VS) MRA has been recently introduced as a promising NCE angiography technique [11,12]. At the core of this method lies VS magnetization preparation, which suppresses stationary tissues and slow-moving venous blood while preserving fast-flowing arterial blood based on velocity. VS preparation can be combined with 3D encoding to enable a high spatial resolution in all three dimensions, unlike 2D multi-slice approaches such as 2D TOF and QISS. The VS preparation directly generates angiographic contrast without relying on the inflow effects and therefore relaxes the requirement of a small imaging thickness. Due to the use of many RF and gradient pulses, the major issue with VS preparation is the sensitivity to B_0_ and B_1_ field errors, which result in arterial signal loss, stripe artifacts, and a non-uniform background suppression. These issues have been addressed by several strategies applied in VS pulse design, including phase-cycled multi-refocusing schemes, periodic shifting of spatial responses, and numerically optimized excitation pulses [13,14,15,16]. While it was developed most actively for peripheral angiography with clinical validation in patients [17], VS-MRA has been shown to be feasible for various vascular beds, including the cerebral, renal, abdominal, and pedal arteries, but not yet for the carotid arteries [11,15,18,19].

In this study, we aimed to optimize VS-MRA protocols for whole-neck angiography at 3T and demonstrate its feasibility in vivo. We first measured B_0_ and B_1_ field maps and vascular flow velocity to determine the structure of pulse sequences and the design parameters for VS pulses. Based on these preliminary measurements, we used pre-compensated VS pulses to mitigate the effects of field offsets and an additional SS preparation to improve the suppression of venous blood. We tested the resultant whole-neck VS-MRA protocol in healthy volunteers and compared its performance with that of clinical 3D TOF.

## 2. Materials and Methods

### 2.1. Preliminary In-Vivo Experiments

Three-dimensional B_0_ and B_1_ field mappings and 2D phase-contrast (PC) flow imaging were performed on five healthy volunteers (23.2 ± 0.84 years of age; one male, four females) to help design VS preparation pulses and scan protocols. A 3T whole-body MR scanner (Tim-TRIO; Siemens Medical Solutions, Erlangen, Germany), and a 16-channel head and neck coil, combined with a 4-channel flex coil, were used. Each field mapping and flow imaging was performed twice at an inferior offset of 48.5 mm (station 1) and a superior offset of 65 mm (station 2) from the iso-center that was positioned right below the carotid bifurcation. This two-station protocol assumes an entire S-I coverage of 232 mm, covering the basilar artery through the subclavian arteries.

3D B_0_ maps were obtained using two GRE acquisitions with an echo delay of 1.0 ms. Other parameters were field of view (FOV) = 220 × 220 × 102/130 (station 1/station 2) mm^3^, spatial resolution = 2.75 × 3.24 × 4.16 mm^3^, echo time/repetition time (TE/TR) = 3.2 or 4.2/8.6 ms, and flip angle = 12°. The 3D B_1_ maps were obtained using the Bloch–Siegert phase-based method with an 8-ms Fermi saturation pulse [20]. Other parameters were FOV = 220 × 220 × 102/130 (station 1/station 2) mm^3^, spatial resolution = 2.75 × 6.88 × 4.2 mm^3^, TE/TR = 4.5/200 ms, and flip angle = 14°. The parameters for the 2D PC flow MRI included VENC = 120/90 (station 1/station 2) cm/s, spatial resolution = 1.04 × 1.48 mm^2^, temporal resolution = 20.05 ms, FOV = 200 × 200 mm^2^, TE/TR = 4.91/20.05 ms, bandwidth = 185 Hz/pixel, and flip angle = 10°.

In the acquired B_0_ and B_1_ maps, polygonal regions of interest (ROIs) were manually specified on major arteries (common carotid, subclavian, and internal carotid arteries) and on nearby muscles to calculate the ranges of field errors for each station. The outliers of 5% were eliminated for each measurement. Similarly, in the PC flow data, ROIs were specified on the carotid arteries and jugular veins to obtain time-velocity curves during cardiac phases. These measurements were then used to calculate the timings of the onset and peak of systolic flow and the velocity values of the arteries and veins of interest at peak systole.

### 2.2. VS-MRA Pulse Sequence

**Figure 1:**
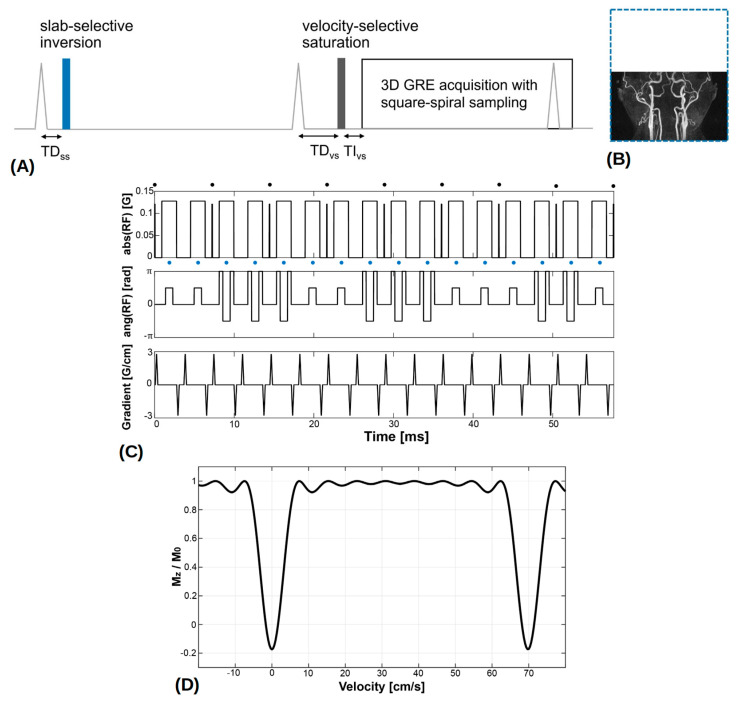
(**A**) Neck velocity-selective magnetic resonance angiography (VS-MRA) pulse sequence was triggered by ECG signals and played a slab-selective (SS) inversion preparation pulse at the onset of systolic flow. The thickness of the SS inversion preparation (blue dotted line) was larger than the imaging volume in the superior direction to encompass upstream venous flow (**B**). After the 2nd ECG triggering, a VS saturation preparation pulse was played near the time of peak systolic flow, followed by a fat saturation pulse and a segmented 3D GRE acquisition. (**C**) VS saturation pulse consisted of nine hard RF sub-pulses (denoted by black circles) interleaved with unipolar gradients for velocity encoding and 90°_x_−180°_y_−90°_x_ composite pulses (denoted by blue circles) for refocusing. (**D**) Simulated Mz response over velocity, resulting from the VS preparation pulse where positive velocity represents the direction of arterial flow. (Abbreviation: TD = trigger delay; TI = inversion time).

Figure 1A shows a schematic view of the VS-MRA pulse sequence for neck angiography. As the preliminary PC flow studies showed that the venous flow velocity was too high to be suppressed by the VS preparation alone (see more details in Results Section 3.1), an adiabatic SS inversion preparation was applied prior to the VS preparation to pre-suppress venous blood, as in a recent study on cerebral VS-MRA [15]. The SS inversion pulse had a thickness larger than the imaging volume in the superior direction by an amount of 16.2/11.5 cm (station 1/station 2) to encompass upstream venous blood (Figure 1B), and was temporally synchronized to the onset of systolic flow through ECG gating to maximize arterial inflow. The adiabatic pulse was 30-ms long, based on the hyperbolic secant envelope function with design parameters of β = 300 rad/s, μ = 14, and bandwidth = 1338 Hz [21].

After the second ECG triggering with a trigger delay for the VS preparation (TD_vs_), a VS saturation preparation pulse was played near the time of peak systolic flow to maximize the velocity difference between arterial blood and background tissues, followed by a spectral-selective fat saturation pulse and a segmented 3D GRE acquisition. Note that, with the use of two ECG triggerings, the imaging volume would experience two systolic inflows of the upstream arterial blood not affected by the SS inversion. The VS saturation pulse was based on the double-refocused design with 90°_x_−180°_y_−90°_x_ composite pulses for refocusing [13], and designed with a flip angle of 100°, nine hard RF sub-pulses, and a velocity FOV of 70 cm/s, resulting in a cut-off velocity of 3.5 cm/s (Figure 1C,D). Based on the prior field error measurements, pre-compensation was applied to VS pulse design by modulating the amplitude (based on B_1_ offsets) and phase (based on B_0_ offsets) of the B_1_ field waveform:

(1) $$B_{1}^{comp}\left(t\right)=AB_{1}\left(t\right)e^{j2\pi ft}$$

where A and f represent the inverse of the measured B_1_ scale and off-resonant frequency, respectively. To suppress stripe artifacts induced by refocusing errors in VS excitation, four VS pulses with spatially shifted excitation profiles were alternately applied, as proposed previously [16].

**Figure 2:**
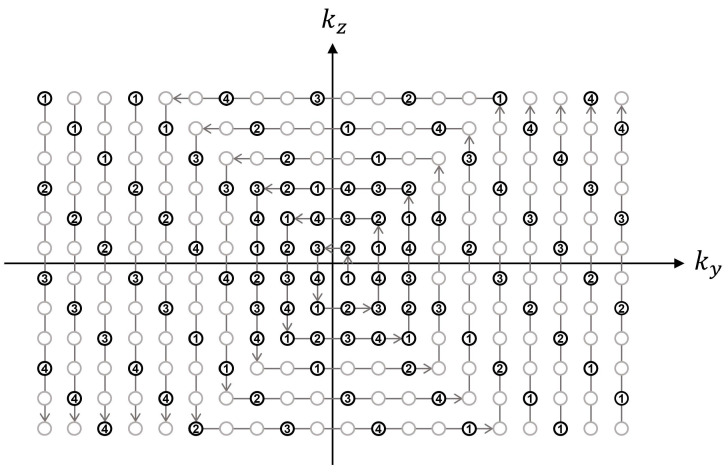
Illustration of square-spiral sampling trajectory in k_y_-k_z_ space, assuming an acceleration factor = 3, self-calibration size = 6 × 6, and views per segment = 26. The sampling position was first determined by traversing the k_y_-k_z_ plane in a square-spiral fashion (denoted by gray arrows) while fully sampling the inner region and three-fold under-sampling the outer region. The gray and black circles represent skipped and acquired points, respectively. The resultant sequence of (k_y_, k_z_) locations was then sequentially segmented for each segmented acquisition (denoted by numbers) where the segmentation factor was determined as the total number of samples divided by the number of views to be acquired for each acquisition.

The segmented acquisition used three-fold k-space under-sampling with the inner k-space fully sampled for a self-calibrated parallel imaging reconstruction [22]. The sampling position in the k_y_-k_z_ space was scheduled in a square-spiral fashion starting at the origin and ending at the periphery of k-space. In this way, center-out k-space weighting could be achieved along both k_y_ and k_z_ dimensions with flexible choices for the number of views to be acquired for each segmented acquisition (Figure 2).

### 2.3. MRA Experiments and Analysis

In-vivo MRA experiments were performed on a 3T clinical whole-body MR scanner (Tim-TRIO; Siemens Medical Solutions, Erlangen, Germany). A 16-channel head and neck coil combined with a 4-channel flex coil were used for signal reception. Seven healthy subjects were scanned after written consent forms, approved by our institutional review board, were obtained. On one subject (46 years old male), B_0_ and B_1_ mappings, and neck VS-MRA using VS preparation pulses with and without the field error compensation, were performed to investigate the effects of field errors and pre-compensation on the performance of VS preparations. On six subjects (29 ± 4.98 years of age; four males, two females), the proposed neck VS-MRA and clinical 3D TOF were performed for comparison.

The VS-MRA protocol used two 3D scans, which contiguously covered the whole-neck arterial tree with a total S-I coverage of 232 mm (same as the coverage of B_0_ and B_1_ mapping described in Section 2.1). The trigger delay for SS preparation (TD_ss_) was set to 40 and 70 ms for the inferior volume (station 1) and the superior volume (station 2), respectively, based on prior PC flow measurements to ensure that the SS preparation can be synchronized to the beginning of systolic flow. Similarly, the TD_vs_ was set to 110/140 ms (station 1/station 2) to be synchronized to peak systolic flow. Other imaging parameters for VS-MRA were TI = 100 ms, spatial resolution = 0.92 × 0.92 × 1.44 mm^3^, FOV for station 1 = 220 × 220 × 102 mm^3^, FOV for station 2 = 220 × 220 × 130 mm^3^, TE/TR = 4.6/7.6 ms, bandwidth = 310 Hz/pixel, flip angle = 14°, three-fold iterative self-consistent parallel imaging (SPIRiT) with a 24 self-calibration size [22], repetition time = 3R-R intervals, views per segment = 71, and scan time = 4.5/5.25 min (station 1/station 2), assuming a heart rate of 70 bpm. For 3D TOF, clinical protocols routinely used in our institution were used with slight modifications, including spatial resolution = 0.86 × 0.86 × 1.44 mm^3^, FOV = 220 × 220 × 232 mm^3^, TE/TR = 3.4/20 ms, bandwidth = 186 Hz/pixel, flip angle = 15°, GRAPPA factor = 2 with reference lines = 36, number of slabs = 8, slices per slab = 28, partial Fourier factor = 6/8 (in both k_y_ and k_z_ directions), and scan time = 9.8 min.

A quantitative analysis of VS-MRA and 3D TOF images was performed by calculating the relative contrast ratio (CR) between artery and muscle signals, defined as (S_A_ − S_M_)/S_A_, where S_A_ and S_M_ are the signal intensities of arterial blood and muscle, respectively [13,23]. The contrast-to-noise ratio (CNR) was not considered due to the spatially varying noise involved in parallel-imaging reconstructed images [24,25]. The relative CR was calculated on twenty major arterial segments (1: brachiocephalic artery; 2 and 3: bilateral subclavian arteries; 4 and 5: bilateral common carotid arteries; 6 and 7: bilateral proximal internal carotid arteries; 8 and 9: bilateral cervical segments of the internal carotid arteries; 10 and 11: bilateral petrous segments of the internal carotid arteries; 12 through 19: V1, V2, V3, and V4 segments of the vertebral arteries; 20: basilar artery). The relative CR of the V4 segment was calculated for only four subjects since this segment was invisible in data obtained from the other two subjects. The relative CRs of the bilateral petrous segments and the basilar artery were calculated only in five subjects’ data since these segments were not included in one subject due to the suboptimal prescription of the imaging FOV. Arterial ROIs of polygonal shapes were manually specified on equally spaced (1.5 mm) axial slices of 3D reconstructed images, and each ROI was copied to two neighboring slices. Similarly, muscle ROIs were placed near the arterial segments. The mean intensities of all pixels in each artery and muscle ROIs were used as S_A_ and S_M_, respectively. A two-sample *t*-test with a two-tailed distribution was used for the statistical test.

## 3. Results

### 3.1. PC Flow and Field Mappings

The timing for the beginning of systolic flow in the carotid arteries was determined as 58.15 ± 9.82 ms for station 1 and 90.23 ± 12.68 ms for station 2, while the timing for the maximum systolic flow was measured as 126.32 ± 15.00 ms for station 1 and 150.38 ± 25.36 ms for station 2. Accordingly, the cardiac TD_ss_ and TD_vs_ for VS-MRA were set to 40/70 ms (station 1/station 2) and 110/140 ms (station 1/station 2), respectively. As the peak flow velocities of carotid arteries were measured as 66.18 ± 4.79 cm/s/46.88 ± 4.30 cm/s (station 1/station 2), a velocity FOV of 70 cm/s was used when designing VS preparation pulses. The velocities of jugular veins were measured as 9.45 ± 7.84 cm/s/18.01 ± 1.84 cm/s (station 1/station 2), which are not included in the stop-band of the VS preparation and justify the use of an additional SS inversion preparation pulse. According to the field map analysis, off-resonance ranged from −145.3 to 346.9 Hz for station 1 and from −202.7 to 97.5 Hz for station 2, while the B_1_ scale ranged from 0.57 to 1.17 for station 1 and from 0.83 to 1.27 for station 2. Based on these, the pre-compensation for the field offsets was applied for VS pulse designs according to Equation (1) with the following values: A = 1.15^−1^ and f = 100 Hz for station 1; A = 0.95^−1^ and f = 53 Hz for station 2 in Equation (1).

### 3.2. Neck MRA Experiments

**Figure 3:**
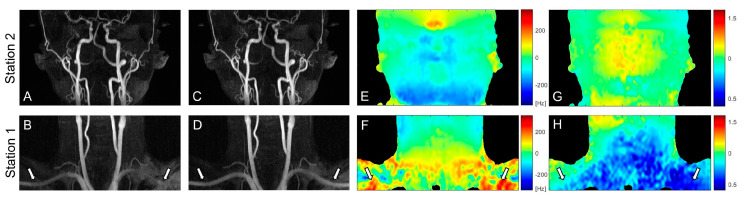
Coronal maximum intensity projections (MIPs) of VS-MRA images obtained using VS preparations without (**A**,**B**) and with pre-compensation (**C**,**D**), along with off-resonance maps (**E**,**F**) and B_1_ maps (**G**,**H**). The original VS preparation suffers from arterial signal loss in the regions of large off-resonance and low B_1_ field, whereas the pre-compensated VS preparation significantly improves the arterial visualization in the same regions (arrows). The images in station 2 (**A**,**C**) show little difference due to relatively low field offsets (**E**,**G**).

Figure 3 contains partial coronal maximum intensity projection (MIP) images of the whole-neck VS-MRA obtained with the original (A, B) or field-error-compensated VS preparation pulses (C, D). B_0_ and B_1_ field maps are also shown in the same subject (E-H). The original VS preparation resulted in signal loss in the subclavian arteries, which involve large magnitudes of B_0_ and B_1_ field errors (arrows). Using the pre-compensated VS preparation, this signal loss was reduced, while the high signal strength was maintained in other arterial branches. Little difference was observed between the images from the two VS preparations in station 2, presumably due to relatively small field offsets (A, C).

**Figure 4:**
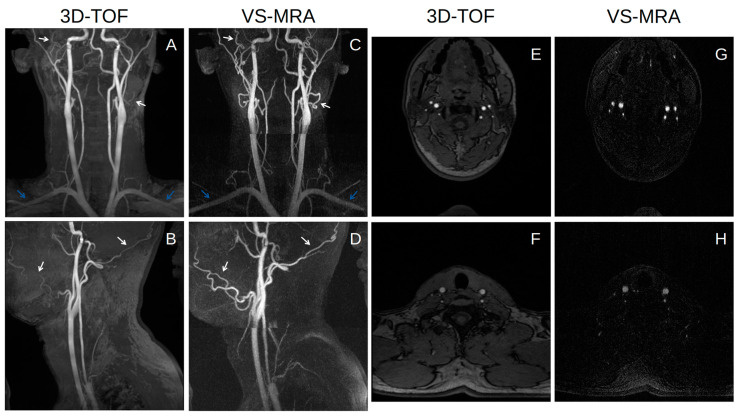
Coronal (**A**,**C**) and sagittal (**B**,**D**) partial MIP images of 3D time-of-flight (TOF) (**A**,**B**) and VS-MRA (**C**,**D**) in a healthy subject, and axial slices in the middle of each station for VS-MRA (**G**,**H**) and in the same locations for 3D-TOF (**E**,**F**). Both 3D TOF and VS-MRA visualize most of the major arteries well over the whole neck. VS-MRA images show improved visualization of small vessels, such as facial and occipital arteries (white arrows), and horizontally oriented arteries, such as subclavian arteries (blue arrows), and also improved the suppression of background signals compared to 3D TOF.

Figure 4 shows representative coronal and sagittal partial MIP images of 3D TOF and VS-MRA performed on a volunteer. Both TOF and VS-MRA successfully visualized most of the major arteries over the neck, except for the V3 and V4 segments of the right vertebral arteries. TOF suffered from signal loss in small distal arteries, such as facial and occipital arteries, likely due to the saturation effect associated with slow flow (white arrows), and in the subclavian arteries, likely due to their in-plane orientation (blue arrows). The depiction of these arteries was improved in VS-MRA due to a higher sensitivity of the VS preparation to slow or in-plane flow. The background signal appeared to be slightly lower in VS-MRA, where VS preparation combined with SS preparation reduced the magnetization of stationary tissues to nearly zero.

**Figure 5:**
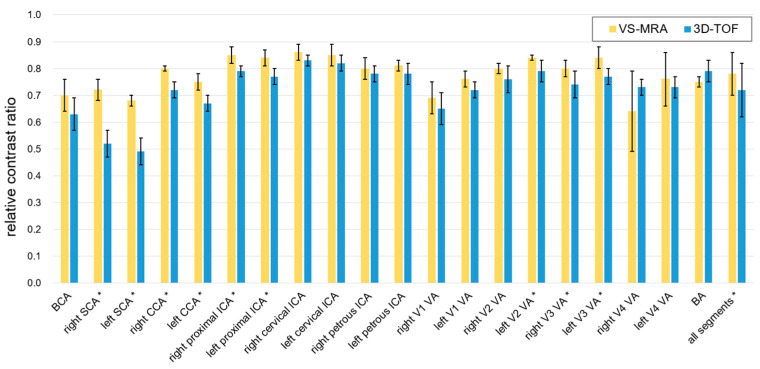
Relative contrast ratio (CR) between artery and muscle signals. Collectively over all 20 arterial segments, the relative CRs were 0.78 ± 0.08 and 0.72 ± 0.10 for VS-MRA and 3D TOF, respectively. The average CR was higher in VS-MRA for all segments, except for the V4 segment of the right vertebral artery and basilar artery. The segments with statistical significance were indicated with asterisks (*p* < 0.05). (Abbreviations: BCA = brachiocephalic artery; SCA = subclavian artery; CCA = common carotid artery; ICA = internal carotid artery; VA = vertebral artery; BA = basilar artery).

The relative artery-to-muscle CR was measured as 0.78 ± 0.08 and 0.72 ± 0.10 for VS-MRA and 3D TOF, respectively, across all twenty arterial segments (*p* < 0.01) (Figure 5). The mean of the relative CR was higher in VS-MRA for all segments (particularly for the bilateral subclavian arteries), except for the V4 segment of the right vertebral and basilar arteries, which is consistent with the images shown in Figure 4.

## 4. Discussion

TOF is a well-established NCE-MRA method, routinely used for neurovascular applications. Based on relatively simple gradient-echo-based pulse sequences, TOF is robust to MR system errors, such as B_0_ and B_1_ field inhomogeneity, and eddy currents. Still, TOF has well-known limitations in visualizing small and/or in-plane-oriented vessel segments, which experience excessive excitations and therefore become saturated as in stationary tissues. These issues turned out to be mitigated in VS-MRA, which allows for higher sensitivity to slow flow through VS saturation preparation. This finding is consistent with a recent study on cerebral VS-MRA compared with 3D TOF [15].

Primary design parameters for VS saturation preparation pulses include the number of hard RF pulses and velocity FOV. Using more hard RF pulses narrows the excitation bandwidth (i.e., the width of the velocity stop-band) and therefore widens the velocity pass-band at the cost of an increased pulse duration. Velocity FOV determines how much excitation profiles are compressed or stretched along the velocity axis. Using larger velocity FOVs increases the upper bound of the velocity pass-band, but also increases the lower bound at the same rate. The cutoff velocity of 3.5 cm/s resulted from nine hard RF pulses, and the velocity FOV of 70 cm/s used in this study appeared to be small enough to highlight slow arterial flow. More than nine hard RF pulses could lower the cutoff velocity while maintaining the upper bound of the velocity passband, but a longer pulse duration will increase the effects of T2 relaxation. A further investigation on these tradeoffs would be warranted in the future.

Although 3D TOF was chosen as the clinical reference in this study, 2D versions are also widely used for clinical neck MRA. The 2D TOF is advantageous for the depiction of slow flow, due to its thin slice thickness which enhances the inflow effect. However, the achievable resolution is limited in the through-plane direction due to the limitation of minimal possible slice thickness (~3 mm), and, in the in-plane direction, due to low SNR. On the other hand, 3D TOF enables a high spatial resolution in all three dimensions and a high SNR, but suffers more from saturation effects, making it difficult to visualize small vessels.

It was necessary to use the SS inversion preparation prior to the VS saturation preparation to suppress venous blood, which was moving faster than the cutoff velocity of the VS preparation. The SS inversion preparation could also improve the suppression of background tissues which were suppressed twice by both SS and VS preparations. However, the additional use of the SS preparation prolonged the scan time compared to the original VS-MRA using VS preparation only. As the original VS-MRA typically uses a repetition time of 1R-R or 2R-R intervals, the scan time penalty factor would be 1.5–3.0 for the 3R-R repetition time used in this study. Higher-rate scan acceleration can be used through advanced compressed sensing, combined with parallel imaging, but at the cost of an SNR penalty. Another limitation of this study is the testing of the proposed VS-MRA in limited numbers of healthy subjects. The performance of the proposed protocol may vary depending on the pattern of arterial flow, particularly in patients with arterial pathologies. This needs to be further investigated in a cohort of patient subjects.

Compared to earlier applications of VS-MRA for peripheral angiography, the increase in the scan time caused by the additional SS preparation is a drawback of the proposed neck VS-MRA. According to our preliminary studies, the use of only the VS preparation with a small velocity FOV resulted in the visualization of both carotid arteries and veins. A promising alternative approach would be to feed these mixed artery-vein images into machine learning algorithms, such as artificial neural networks, to separate the arteries from the veins. In particular, the convolution-based U-net and its variants appear to be promising due to recent great success in various medical image segmentations and syntheses [26,27,28].

## 5. Conclusions

We have developed a VS-MRA protocol for whole neck angiography, which combines adiabatic SS inversion and field-error-compensated VS saturation preparations. By synchronizing the SS and VS preparations to the onset and peak of systolic flow, respectively, the proposed method was able to achieve the successful visualization of the whole neck arterial tree while suppressing stationary tissues and venous blood. VS-MRA yielded relative artery-to-muscle CRs comparable to those obtained with 3D TOF over all arterial segments (0.78 ± 0.08 vs 0.72 ± 0.10) and improved the depiction of small distal or in-plane-oriented arteries, which are challenging to visualize by TOF due to saturation effects.

## Data Availability

The datasets generated and/or analyzed during the current study are available from the corresponding author upon reasonable request.
